# Factors affecting abnormal triggering with non‐invasive ventilators: A before‐and‐after study

**DOI:** 10.1111/crj.13497

**Published:** 2022-05-31

**Authors:** Zhaoning Xu, Dongqin Sheng, Kefan Jiao, Chi Zhang, Junping Hao, Dedong Ma

**Affiliations:** ^1^ School of Nursing and Rehabilitation Shandong University Jinan China; ^2^ The first clinical medical college of Lanzhou University Lanzhou China; ^3^ School of stomatology Shandong University Jinan China; ^4^ Department of Pulmonary and Critical Care Medicine, Qilu Hospital Shandong University Jinan China

**Keywords:** abnormal triggering, auto‐triggering, ineffective‐triggering, non‐invasive ventilation, patient comfort

## Abstract

**Introduction:**

Abnormal triggering of non‐invasive ventilator (NIV) is the main reason for increasing the possibility of patient intolerance and directly affecting the treatment effect.

**Objective:**

To investigate factors that affect abnormal triggering of NIV.

**Methods:**

Thirty health volunteers from August 2018 to August 2019 were recruited. Two kinds of NIVs, Curative Flexo ST30 and Resmed Stellar™ 150, were selected, with S/T mode, respiratory rate of 10 bpm and inspiration time of 1.0 s. Every volunteer received ventilation in two ventilators, five oxygen flows (5/10/15/20/25 L/min), five support pressures (independent inspired positive airway pressure/expired positive airway pressure [IPAP/EPAP]: 8/4; 10/4; 12/4; 16/6; 20/8 cm H_2_O), two oxygen injection sites (proximal to the mask or the ventilator) and three masks (Curative: Bestfit™; Resmed: Mirage Quattro Full Face Mask™; Zhongshan: ZS‐MZ‐A™) for 60 min, respectively.

**Results:**

All factors mentioned above affected normal triggering. With the increase of oxygen flow and support pressure, the frequency of auto‐triggering and ineffective‐triggering increased (*P* < 0.05). For Curative Flexo ST30 ventilator, when the oxygen injection site was proximal to the ventilator, the frequency of auto‐triggering and ineffective‐triggering is significantly lower than when it was proximal to the mask, whereas the result in Resmed Stellar™ 150 is totally different (*P* < 0.05).

**Conclusion:**

When using Curative Flexo ST30 ventilator, the oxygen injection site should be proximal to the ventilator, whereas Resmed Stellar™ 150 ventilator is just the opposite. Attention should be paid to the effectiveness of ventilators when oxygen flow and support pressure settings are high, as abnormal triggers occur more frequently. Choosing the suitable mask is also important.

## Graphical abstract

We investigated several factors which may affect abnormal triggering of two types of non‐invasive ventilators, including oxygen flow, support pressure, oxygen injection site and mask. We mainly concluded that when using Curative Flexo ST30 ventilator, the oxygen injection site should be proximal to the ventilator, while Resmed StellarTM 150 ventilator is just the opposite. Meanwhile, attention should be paid to the effectiveness of ventilators when oxygen flow and support pressure settings are high.

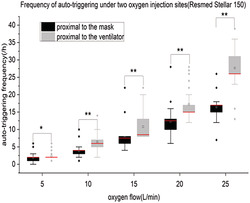

## 1 INTRODUCTION

Non‐invasive ventilation (NIV) is popular in clinical practice due to its ability to reduce tracheal intubation, intensive care unit length (ICU) and mortality^1^ and is an effective treatment for some forms of respiratory failure due to many diseases, such as chronic obstructive pulmonary disease (COPD), cardiogenic pulmonary edema (CPE), obesity hypoventilation and post‐extubation respiratory failure.^2^

The basic operational sequence of the NIV with pressure‐control mode is triggering, rate of pressurization, level of pressure support, inspiration‐expiration cycling and expired positive airway pressure (EPAP) level.^3^ Active inspiration by patients can cause pressure and flow fluctuations, which are transmitted to sensors that trigger the inspiratory process of the NIV. This is normal triggering.^4^ Patient–ventilator asynchrony can be defined as a mismatch between the patient and ventilator inspiratory and expiratory times.^5^, ^6^ Patient–ventilator asynchrony can arise in all phases.^7^ However, the incidence of problems is higher during the inspiratory trigger phase.^8^

Patient–ventilator asynchronies have been reported to occur with a high incidence during NIV in critically ill patients, reaching 43%,^9^ mainly attributed to abnormal triggering. Asynchronies may occur at two levels: during inspiratory triggering when there is a mismatch between patient inspiratory effort and ventilator triggering (i.e., ineffective‐triggering, double‐triggering or auto‐triggering) or during cycling from inspiration to expiration when ventilator cycling does not coincide with the end of patient effort (i.e., premature or delayed cycling).^10^ Auto‐triggering occurs when ventilator initiates two or more cycles at a rate that is higher than the patient's and the back‐up respiratory rate if set.^11^ Ineffective‐triggering occurs when the patient's inspiratory effort fails to trigger a ventilator breath, resulting in inadequate ventilation for the patient.^10^, ^12^ Asynchrony has been cited as an indirect cause of NIV immediate failure,^13^ which is a major cause of reduced ventilator comfort, significantly increases patient intolerance, and also hinders the recognition of breathing patterns, thus affecting respiratory mechanics.^14^ Patient–ventilator asynchrony can be influenced by a number of factors, including the patient's respiratory system mechanics,^15^ breathing pattern,^10^ drive to breathe,^16^ ventilator settings^15^ and type of interface used.^16^ A good match between patient respiratory efforts and ventilator breaths optimizes patient comfort and reduces work of breathing.^5^

Poor patient–ventilator interaction is common during the NIV with Pressure Support Ventilation (PSV) mode.^17^ Dual positive airway pressure ventilation (bilevel ventilation) provides independent inspired positive airway pressure (IPAP) and EPAP. Most patients who use NIV require supplemental oxygen, but most non‐invasive ventilators are not equipped with an oxygen module for oxygen delivery,^12^ so when using these kinds of ventilators, oxygen is usually connected proximal to the mask or to the ventilator. Few studies have investigated the effects of oxygen injection site, oxygen flow, support pressure and mask on abnormal triggering. Identifying factors that increase the incidence of abnormal triggering may help optimize ventilator settings, minimize patient–ventilator asynchrony and improve patients' comfort.

In order to prospectively and non‐invasively evaluate the incidence of major asynchrony during NIV and further study the different oxygen injection sites, oxygen flows, support pressures (IPAP and EPAP) and masks on the effect of NIV triggering, improve the efficiency of the ventilator and finally achieve the best state of patient–ventilator cooperation, we designed this before‐and‐after study and recruited healthy volunteers using two types of non‐invasive ventilators and recorded the frequency of abnormal triggering under several circumstances.

## 2 MATERIALS AND METHODS

### 2.1 Study population

From August 2018 to August 2019, we recruited 30 healthy medical students from a university in Shandong Province, China. Inclusion criteria are as follows: (I) age range: 18–30 years; (II) normal lung function; (III) no smoking history; and (IV) signed informed consent. Exclusion criteria are as follows: (I) with chronic heart and lung disease or pleural or thoracic diseases; (II) with nosebleed, rhinitis, facial trauma or postoperative deformity; (III) unable to cooperate throughout the process. Written informed consent forms were obtained from all participants.

### 2.2 Study methods

**FIGURE 1:**
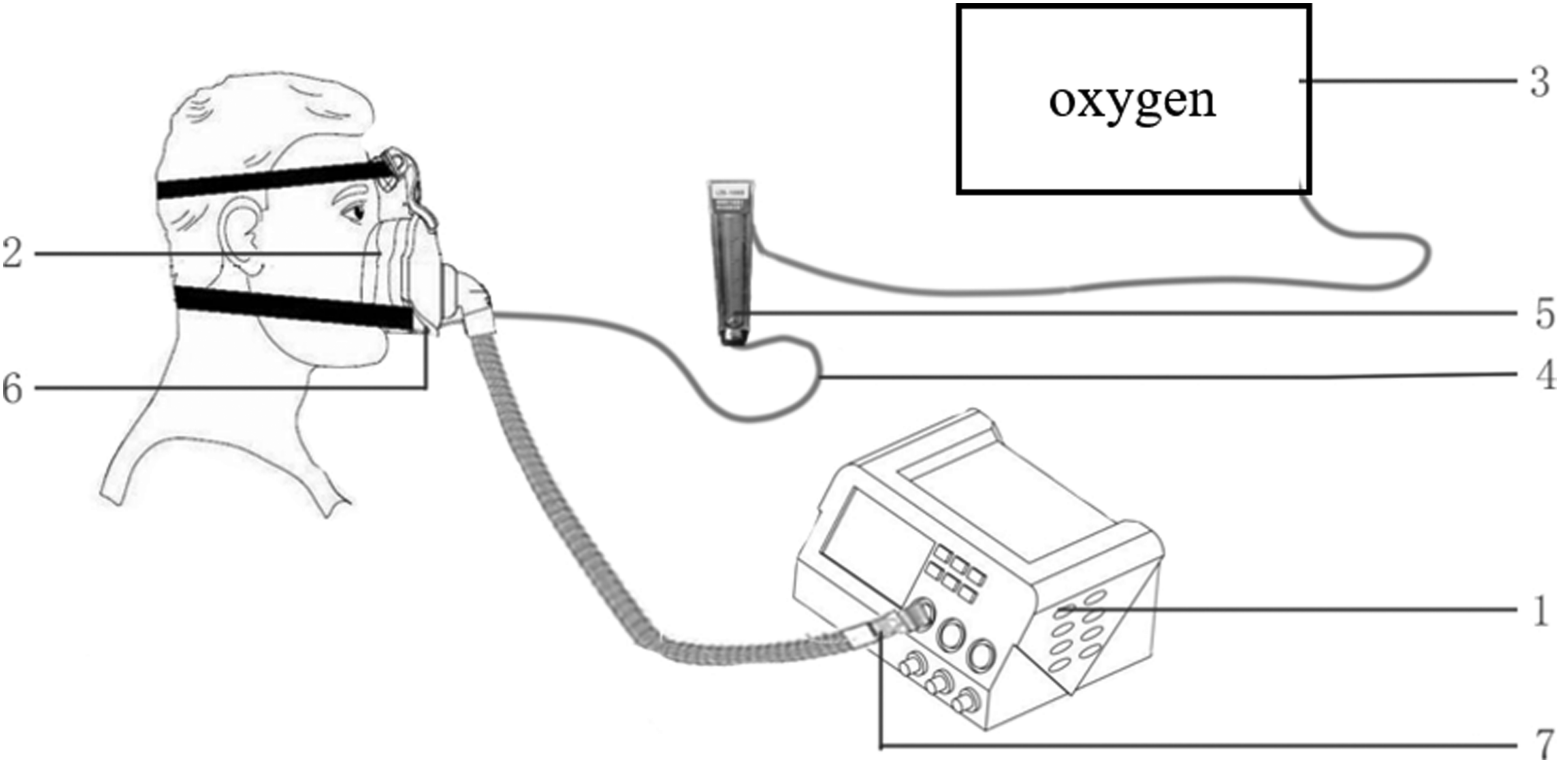
The connection of all devices. 1, non‐invasive ventilator; 2, mask; 3, air compressor; 4, oxygen tube; 5, glass rotor flowmeter (LZB‐10WB™); 6, oxygen injection site: proximal to the mask; 7 oxygen injection site: proximal to the ventilator

This is a before‐and‐after study. All volunteers were first informed of the possible discomfort associated with the use of ventilators and that this temporary discomfort would not affect their health in order to gain their cooperation. The doors and windows of the room where the study was conducted were well closed and equipped with air conditioning to maintain a constant room temperature (24–26°C). The flow of oxygen was controlled by the glass rotor flowmeter (LZB‐10WB™). Oxygen is injected through the sites marked with the numbers 6 or 7, respectively, in Figure 1. Two kinds of non‐invasive ventilators, Curative Flexo ST30 and Resmed Stellar™ 150, were selected for this study, with PSV, S/T mode, the respiratory rate of 10 bpm and an inspiration time of 1.0 s. The connection of above devices is shown in Figure 1.

Every volunteer first sat for 10 min to reach a stable breathing state and first breathed for 60 min using each ventilator at two oxygen injection points and five different oxygen flow rates (5, 10, 15, 20 and 25 L/min) with the same pressure support level (IPAP = 10 cm H_2_O and EPAP = 4 cm H_2_O). Then, every volunteer breathed using each ventilator for 60 min at two oxygen injection sites and five different pressure support levels (IPAP = 8 cm H_2_O, EPAP = 4 cm H_2_O; IPAP = 10 cm H_2_O, EPAP = 4 cm H_2_O; IPAP = 12 cm H_2_O, EPAP = 4 cm H_2_O; IPAP = 16 cm H_2_O, EPAP = 6 cm H_2_O; IPAP = 20 cm H_2_O, EPAP = 8 cm H_2_O), respectively. Thirdly, every volunteer breathed for 60 min using each ventilator at two oxygen injection points, five different oxygen flow rates (5, 10, 15, 20 and 25 L/min) and three different masks (Curative: Bestfit™; Resmed: Mirage Quattro Full Face Mask™; Zhongshan: ZS‐MZ‐A™) with the same pressure settings (IPAP = 10 cm H_2_O and EPAP = 4 cm H_2_O). Meanwhile, there was a 24 h washout period between each setting trial.

**FIGURE 2:**
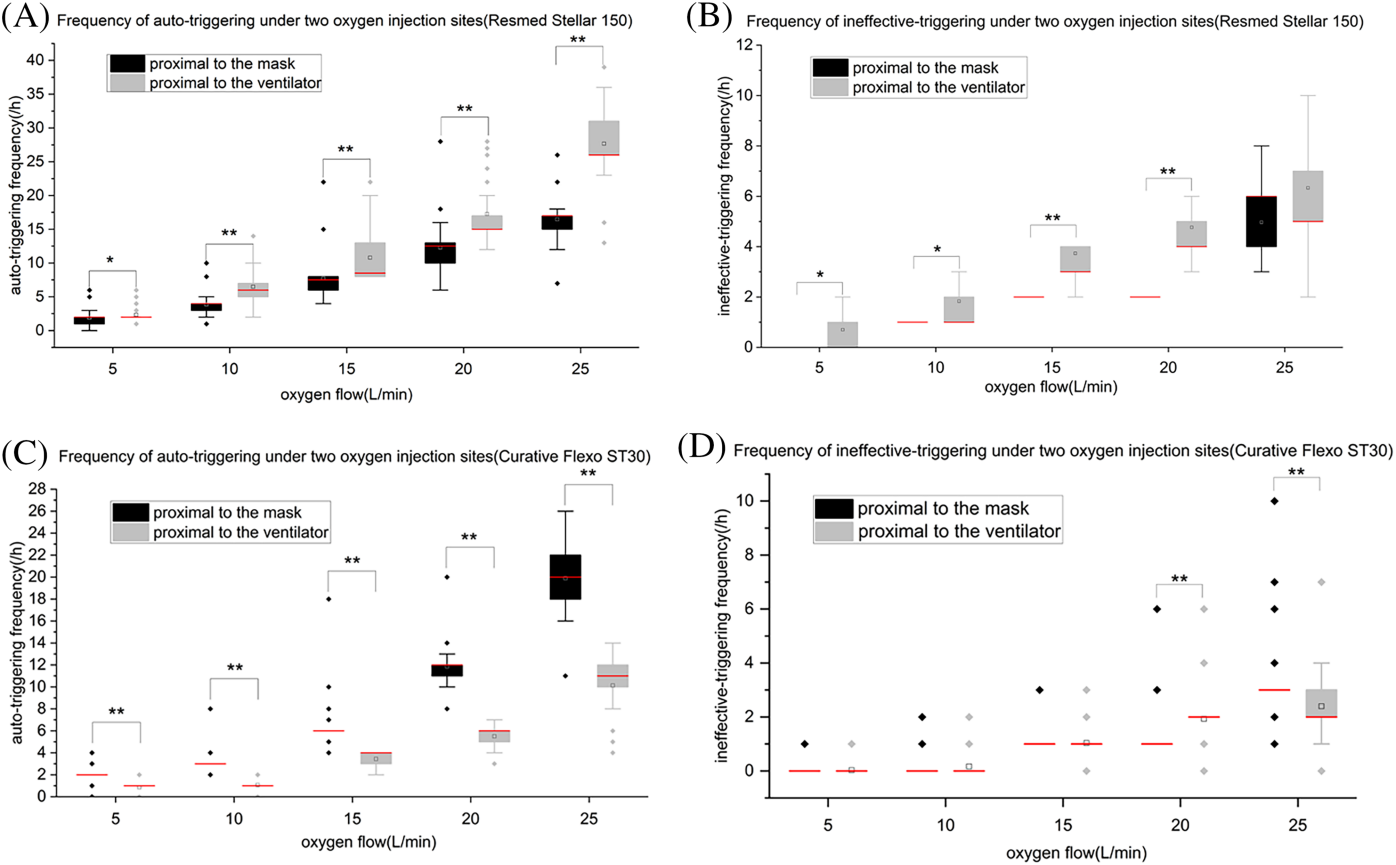
Frequency of auto‐triggering and ineffective‐triggering under two oxygen injection sites, five oxygen flows in two ventilators. *P < 0.05, **P < 0.001. Figure 2A,B shows the results of the frequency of auto‐triggering and ineffective‐triggering of Resmed stellar™ 150 under two oxygen injection sites using five oxygen flows, respectively. Figure 2C,D shows the results of the frequency of auto‐triggering and ineffective‐triggering of curative Flexo ST30 under two oxygen injection sites using five oxygen flows, respectively

**FIGURE 3:**
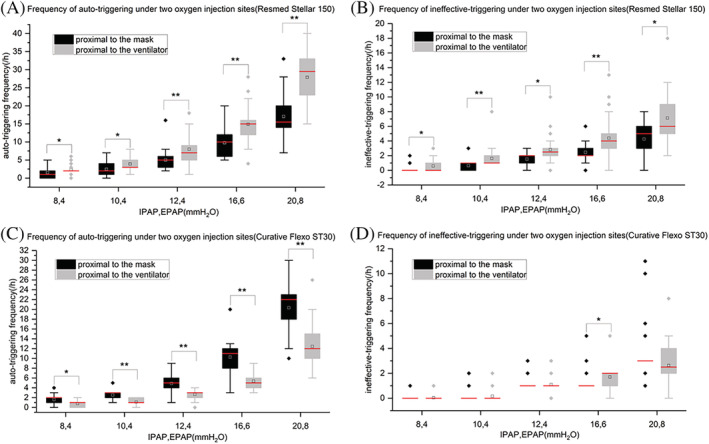
Frequency of auto‐triggering and ineffective‐triggering under two oxygen injection sites, five support pressures in two ventilators. *P < 0.05, **P < 0.001. Figure 3A,B shows the results of the frequency of auto‐triggering and ineffective‐triggering of the Resmed stellar™ 150 under two oxygen injection sites using five IPAP and EPAP, respectively. Figure 3C,D shows the results of the frequency of auto‐triggering and ineffective‐triggering of the curative Flexo ST30 under two oxygen injection sites using five IPAP and EPAP, respectively

Finally, all the data were obtained through the SD card of each ventilator. Asynchrony was detected by visual inspection of the recordings from Curative Flexo ST30 and Resmed Stellar™ 150 professional software, Lotus Auto Manager V3_3_1 and Rescan™ software. We investigated patterns of major asynchrony that are easily detected by clinicians, auto‐triggering and ineffective‐triggering. Auto‐triggering was defined as a cycle delivered by the ventilator without a prior airway flow decrease, indicating that the ventilator delivered a breath that was not triggered by volunteers (Figure 2). Ineffective‐triggering was defined as a drop‐in flow due to volunteers' inspiratory effort that does not cause the ventilator delivering a breath (Figure 3). We define ‘frequency’ as the number of abnormal triggering that occurs within an hour. We assessed reproducibility by having two investigators each perform two independent and blinded analyses of all the study recordings. Two chief physicians in the department of respiratory and critical care medicine independently counted the frequency of abnormal triggering. If the volunteers could not tolerate or voluntarily asked to stop the study, the process was terminated.

The trial was approved by the Medical Ethics Committee of Qilu Hospital of Shandong University (No. 2018216) and registered in the Chinese Clinical Trials Registry (ChiCTR2100053834).

### 2.3 Statistical analysis

SPSS version 22.0 (SPSS Inc., Chicago, IL, USA) was used for data processing and analysis. When the measurement data were not normally distributed, the median (interquartile range) [M (P25, P75)] was used instead of the mean ± standard deviation. The Mann–Whitney *U* test was used to compare the non‐normal measurements. Results were compared between multiple groups using the Kruskal–Wallis rank sum test. To evaluate the validity of the method, we used the κtest. *P* < 0.05 was considered statistically significant.

## 3 RESULTS

### 3.1 Baseline characteristics

A total of 30 volunteers were eligible for inclusion from August 2018 to August 2019. All the volunteers completed the whole process, and there are no important harms or unintended effects in all volunteers. The characteristics of these 30 volunteers are summarized in Table 1. The κtest comparing the ability of the two observers to detect patients with asynchrony showed very high agreement, with aκvalue of 0.96 (*P* < 0.01).

**TABLE 1:** Baseline characteristics

Variable	Total	Male	Female
Frequency	30	12	18
Age	26.1 ± 2.9	25.8 ± 0.8	26.3 ± 3.7
Height/cm	167.7 ± 7.3	176.0 ± 1.6	162.2 ± 3.1
Weight/kg	64.6 ± 15.8	82.7 ± 4.0	52.7 ± 5.8
BMI/kg * m^−2^	22.7 ± 3.6	26.7 ± 1.0	20.0 ± 1.7

Abbreviation: BMI, body mass index.

### 3.2 Outcomes

#### 3.2.1 Frequency of auto‐triggering and ineffective‐triggering of two kinds of ventilators under five different oxygen flows and two different oxygen injection sites

Under the same support pressure (IPAP = 10 cm H_2_O, EPAP = 4 cm H_2_O), the frequency of auto‐triggering and ineffective‐triggering occurred when the oxygen was added proximal to the mask for the Resmed Stellar™ 150 ventilator under different oxygen flows was statistically significantly lower than that when the oxygen was added proximal to the ventilator (*P* < 0.05) (Figure 1A,B). When the oxygen flow was set to 25 L/min, the comparison of the frequency of ineffective‐triggering showed the opposite results, but the difference was not statistically significant (*P* > 0.05). Meanwhile, with the increase of oxygen flow (2 L/min), the frequency of auto‐triggering and ineffective‐triggering in both oxygen injection sites tended to increase, and most of the difference was statistically significant (*P* < 0.05) (Table 2).

**TABLE 2:** Frequency of auto‐triggering and ineffective‐triggering of Resmed stellar™ 150 and curative Flexo ST30 ventilator under 5 different oxygen flows

Oxygen flow (L/min)	Frequency of AT (R)		Frequency of IT (R)		Frequency of AT (C)		Frequency of IT (C)	
	Mask^#^	Ventilator^#^	Mask^#^	Ventilator^#^	Mask^#^	Ventilator^#^	Mask^#^	Ventilator^#^
5	2 (1–2)^‡,§,¶,※^	2 (2–2.25)^‡,§,¶,※^	0 (0–0)^‡,§,¶,※^	0 (0–1)^‡,§,¶,※^	2 (2–2)^§,¶,※^	1 (1–1)^§,¶,※^	0 (0–0)^§,¶,※^	0 (0–0)^§,¶,※^
10	4 (3–4)^†,§,¶,※^	6 (5–7)^†,§,¶,※^	1 (1–1)^†,§,¶,※^	1 (1–2)^†,§,¶,※^	3 (3–3)^§,¶,※^	1 (1–1)^§,¶,※^	0 (0–0)^§,¶,※^	0 (0–0)^§,¶,※^
15	7.5 (6–8)^†,‡,¶,※^	8.5 (8–13.25)^†,‡,¶,※^	2 (2–2)^†,‡,※^	3 (2.75–4)^†,‡,※^	6 (6–6.25)^†,‡,¶,※^	4 (3–4)^†,‡,¶,※^	1 (1–1)^†,‡,※^	1 (1–1)^†,‡,¶,※^
20	12.5 (10–13)^†,‡,§,※^	15 (15–17.75)^†,‡,§,※^	2 (2–2.25)^†,‡,※^	4 (4–5)^†,‡^	12 (10.75–12)^†,‡,§,※^	6 (5–6)^†,‡,§,※^	1 (1–1)^†,‡,※^	2 (2–2)^†,‡,§^
25	17 (15–17)^†,‡,§,¶^	26 (26–31)^†,‡,§,¶^	6 (3.75–6)^†,‡,§,¶^	5 (5–7)^†,‡,§^	20 (18–22)^†,‡,§,¶^	11 (9.5–12)^†,‡,§,¶^	3 (3–3)^†,‡,§,¶^	2 (2–3)^†,‡,§^

*Note*: mask^#^ indicates “proximal to the mask”; ventilator^#^ indicates “proximal to the ventilator’; AT indicates ‘auto‐triggering’; IT indicates ‘ineffective triggering’; R indicates ‘Resmed Stellar™ 150 ventilator’; C indicates ‘Curative Flexo ST30 ventilator’; data were shown as IQR (interquartile range) [M (P25, P75)]. †, ‡, §, ¶ and ※ indicate that the comparison within the same oxygen injection and same kind of triggering group was statistically significant compared with oxygen flow is 5, 10, 15, 20 and 25 L/min (*P* < 0.05) respectively.

Under the same support pressure (IPAP = 10 cm H_2_O, EPAP = 4 cm H_2_O), the frequency of auto‐triggering that occurred when the oxygen was added proximal to the mask for the Curative Flexo ST30 ventilator under different oxygen flows was statistically significantly higher than that when the oxygen was added proximal to the ventilator (*P* < 0.05) (Figure 2C). There is no statistically significant difference in ineffective‐triggering times between two oxygen injection positions (Figure 2D). Meanwhile, with the increase of oxygen flow, the frequency of auto‐triggering and ineffective‐triggering in both oxygen injection sites tended to increase, and most of the difference was statistically significant (*P* < 0.05) (Table 2).

#### 3.2.2 Frequency of auto‐triggering and ineffective‐triggering of two kind of ventilators under five different pressure supports (IPAP and EPAP) and two different oxygen injection sites

For the Resmed Stellar™ 150 ventilator, under the same oxygen flow rate (2 L/min) and different pressure support, the frequency of auto‐triggering and ineffective‐triggering occurred when the oxygen was added proximal to the mask was statistically significantly lower than that when the oxygen was added proximal to the ventilator (*P* < 0.05) (Figure 3A,B). Meanwhile, with the increase of pressure support (IPAP and EPAP), the frequency of auto‐triggering and ineffective‐triggering in both oxygen delivery positions tended to increase, and most of the difference was statistically significant (*P* < 0.05) (Table 3).

**TABLE 3:** Frequency of auto‐triggering and ineffective‐triggering of Resmed stellar™ 150 and curative Flexo ST30 ventilator under 5 different support pressures (IPAP, EPAP)

IPAP, EPAP (mmHg)	Frequency of AT (R)		Frequency of IT (R)		Frequency of AT (C)		Frequency of IT (C)	
	Mask^#^	Ventilator^#^	Mask^#^	Ventilator^#^	Mask^#^	Ventilator^#^	Mask^#^	Ventilator^#^
8, 4	1 (0–2)^§,¶,※^	2 (2–2.25)^§,¶,※^	0 (0–0)^§,¶,※^	0 (0–1)^‡,§,¶,※^	2 (1–2)^§,¶,※^	1 (0–1)^§,¶,※^	0 (0–0)^§,¶,※^	0 (0–0)^§,¶,※^
10, 4	2 (1–4)^§,¶,※^	3 (2.75–5)^§,¶,※^	1 (0–1)^§,¶,※^	1(1–2)^a,c,d,e^	3 (2–3)^§,¶,※^	1 (1–2)^§,¶,※^	0 (0–0)^§,¶,※^	0(0–0)^§,¶,※^
12, 4	5 (3–6)^†,‡,¶,※^	7 (5–9.25)^†,‡,¶,※^	2 (1–2)^†,‡,¶,※^	2.5 (2–3)^†,‡,¶,※^	5 (4–6)	3 (2–3)	1(1–1)^†,‡,※^	1 (1–1)^†,‡,※^
16, 6	10 (6–12.25)^†,‡,§,※^	15 (12–16.25)^†,‡,§,※^	2 (2–3)^†,‡,§^	4 (3–5)^†,‡,§,※^	11 (8–12)	5 (4–6)	1 (1–1)^†,‡,※^	2 (1–2)^†,‡^
20, 8	15.5 (14–20)^†,‡,§,¶^	29.5 (22.75–33)^†,‡,§,¶^	5 (3–6)^†,‡,§^	6 (5–9)^†,‡,§,¶^	22 (17.5–23.25)	12 (9.75–15)	3 (3–3)^†,‡,§,¶^	2.5 (2–4)^†,‡,§^

*Note*: mask^#^ indicates ‘proximal to the mask’; ventilator^#^ indicates ‘proximal to the ventilator’; AT indicates ‘auto‐triggering’; IT indicates ‘ineffective triggering’; R indicates ‘Resmed Stellar™ 150 ventilator’; C indicates ‘Curative Flexo ST30 ventilator’; data were shown as IQR (interquartile range) [M (P25, P75)]. †, ‡, §, ¶ and ※ indicate that the comparison within the same oxygen injection and same kind of triggering group was statistically significant compared with IPAP and EPAP is 8 and 4 cm H_2_O, 10 and 4 cm H_2_O, 12 and 4 cm H_2_O, 16 and 6 cm H_2_O and 20 and 8 cm H_2_O (*P* < 0.05), respectively.

Abbreviations: EPAP, expired positive airway pressure; IPAP, independent inspired positive airway pressure.

Under the same oxygen flow rate (2 L/min), the frequency of auto‐triggering occurred when the oxygen was added proximal to the mask for the Curative Flexo ST30 ventilator under different pressure support was statistically significantly higher than that when the oxygen was added proximal to the ventilator (*P* < 0.05) (Figure 3C). There was no significant difference in the frequency of ineffective‐triggering for different oxygen delivery positions (Figure 3D). Meanwhile, with the increase of pressure support (IPAP and EPAP), the frequency of auto‐triggering and ineffective‐triggering in both oxygen delivery positions tended to increase, and most of the difference was statistically significant (*P* < 0.05) (Table 3).

#### 3.2.3 Frequency of auto‐triggering and ineffective‐triggering of two kind of ventilators under three different masks and two different oxygen injection sites

**FIGURE 4:**
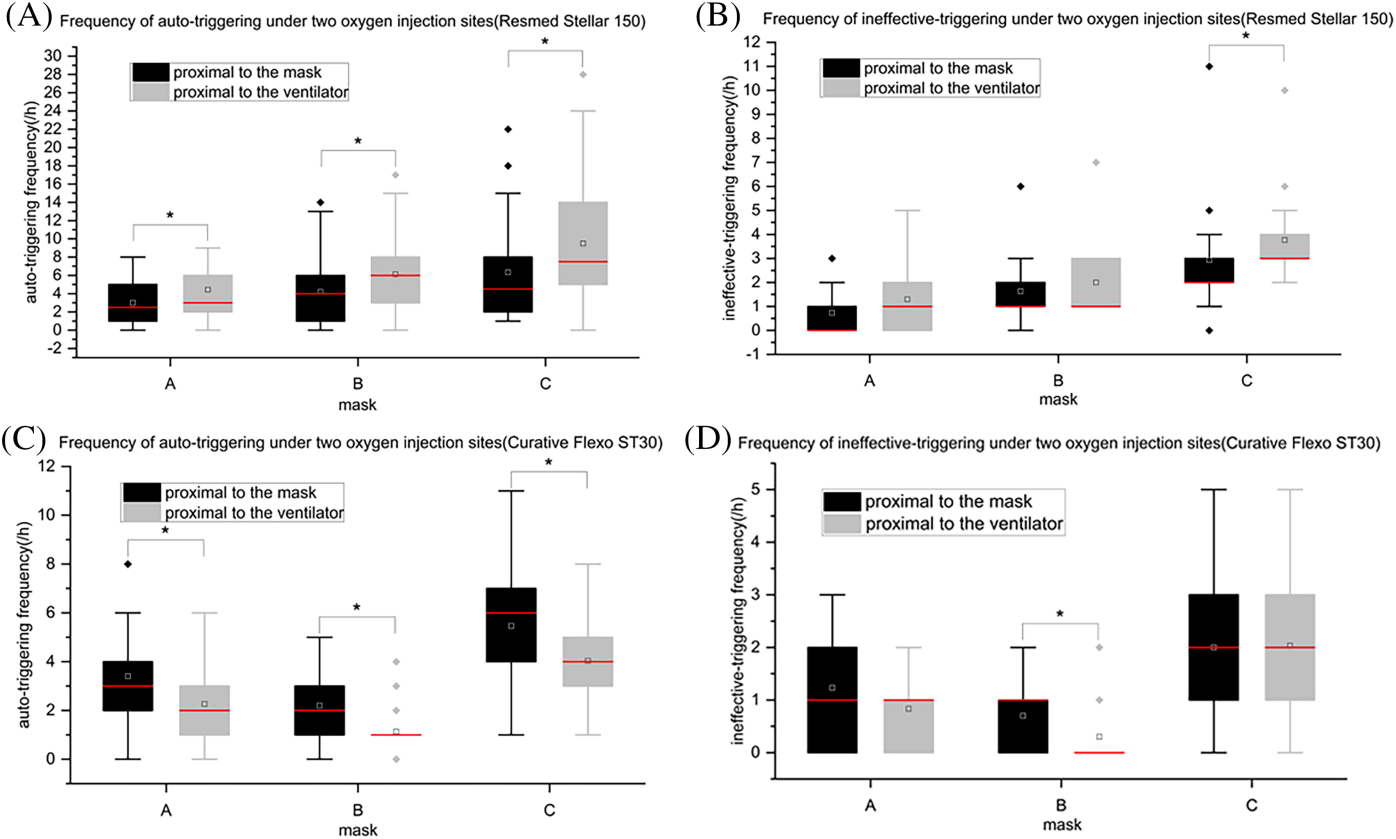
Frequency of auto‐triggering and ineffective‐triggering under two oxygen injection sites, three masks in two ventilators. *P < 0.05, **P < 0.001. Figure 4A,B shows the results of the frequency of auto‐triggering and ineffective‐triggering of the Resmed stellar™ 150 under two oxygen injection sites using three kinds of masks, respectively. Figure 4C,D shows the results of the frequency of auto‐triggering and ineffective‐triggering of the curative Flexo ST30 under two oxygen injection sites using three kinds of masks, respectively

Under the same oxygen flow rate (2 L/min) and same support pressure (IPAP = 10, EPAP = 4), the frequency of auto‐triggering occurred proximal to the mask for the Resmed Stellar™ 150 ventilator was statistically significantly lower than that proximal to the ventilator (*P* < 0.05) regardless of mask type (Figure 4A). The frequency of ineffective‐triggering that occurred proximal to the mask was lower than that proximal to the ventilator without statistical significance when using A and B masks, respectively (*P* > 0.05). When using the C mask (Zhongshan: ZS‐MZ‐A), the frequency difference between two oxygen injection sites has statistical significance (*P* < 0.05) (Figure 4B). Meanwhile, for the ResMed Stellar™ 150 ventilator, auto‐triggering and ineffective‐triggering occurred lowest when using the A mask. At both two oxygen injection sites, the frequency of auto‐triggering was statistically lower with the A mask than with the C mask. When administering oxygen proximal to the mask, the frequency of ineffective‐triggering with the A mask was statistically lower than that with the B or C mask. When administering oxygen proximal to the ventilator, the frequency of ineffective‐triggering with the A mask was statistically lower than with the C mask (Table 4).

**TABLE 4:** Frequency of auto‐triggering and ineffective‐triggering of Resmed stellar™ 150 and curative Flexo ST30ventilator under three different masks

Mask	Frequency of AT (R)		Frequency of IT (R)		Frequency of AT (C)		Frequency of IT (C)	
	Mask^#^	Ventilator^#^	Mask^#^	Ventilator^#^	Mask^#^	Ventilator^#^	Mask^#^	Ventilator^#^
A	2.5 (1–5)^§^	3 (2–6.25)^§^	0 (0–1.25)^‡,§^	1 (0–2)^§^	3 (2–4)^‡,§^	2 (1–3)^‡,§^	1 (0–2)^‡,§^	1 (0–1)^‡,§^
B	4 (1–6)	6 (2.75–8.25)	1 (1–2)^†,§^	1 (1–3)^§^	2 (1–3)^†,§^	1 (0.75–1)^†,§^	1 (0–1)^†,§^	0 (0–0)^†,§^
C	4.5 (2–8.25)^†^	7.5 (5–14)^†^	2 (2–3)^†,‡^	3 (3–4)^†,‡^	6 (4–7)^†,‡^	4 (3–5)^†,‡^	2 (1–3)^†,‡^	2 (1–3)^†,‡^

*Note*: mask^#^ indicates ‘proximal to the mask’; ventilator^#^ indicates ‘proximal to the ventilator’; AT indicates ‘auto‐triggering’; IT indicates ‘ineffective triggering’; R indicates ‘Resmed Stellar™ 150 ventilator’; C indicates ‘Curative Flexo ST30 ventilator’; data were shown as IQR (interquartile range) [M (P25, P75)]. ‘A’ indicates ‘Curative: Bestfit™’; ‘B’ indicates ‘Resmed: Mirage Quattro Full Face Mask™’; ‘C’ indicates ‘Zhongshan: ZS‐MZ‐A™’. †, ‡ and § indicate that the comparison within the same oxygen injection and same kind of triggering group was statistically significant compared with mask is A, B and C (*P* < 0.05), respectively.

Under the same oxygen flow rate (2 L/min) and same support pressure (IPAP = 10, EPAP = 4 cm H_2_O), no matter what kind of mask was used, the frequency of auto‐triggering occurred proximal to the mask for Curative Flexo ST30 ventilator was statistically significantly higher than that proximal to the ventilator (*P* < 0.05) (Figure 4C). When using the B mask (Resmed: Mirage Quattro Full Face Mask™), the frequency difference between two oxygen injection sites has statistical significance (*P* < 0.05) (Figure 4D). Meanwhile, for Curative Flexo ST30 ventilator, auto‐triggering and ineffective‐triggering occurred lowest both in two oxygen injection sites when using the B mask (*P* < 0.05) (Table 4).

## 4 DISCUSSION

The major findings of the present study are as follows: (1) The frequency of auto‐triggering and ineffective‐triggering occurred proximal to the ventilator at different oxygen flow rates was significantly higher than that proximal to the mask at the same pressure support (IPAP/EPAP: 10/4 cm H_2_O) as for the Resmed Stellar™ 150 ventilator. The Curative Flexo ST30 ventilator, on the other hand, produced the opposite results; (2) at the same pressure support (IPAP/EPAP: 10/4 cm H_2_O), the frequency of auto‐triggering and ineffective‐triggering of the Resmed Stellar™ 150 and Curative Flexo ST30 ventilator was on the rise as the oxygen flow rate increased, regardless of whether the oxygen injection position was proximal to the mask or proximal to the ventilator; (3) at the same oxygen low rate (2 L/min), the frequency of auto‐triggering and ineffective‐triggering of the Resmed Stellar™ 150 and Curative Flexo ST30 ventilator increased with increasing IPAP and EPAP, regardless of whether the oxygen injection position was proximal to the mask or proximal to the ventilator; (4) using the same brand of mask as the ventilator, the ventilator had fewer auto‐triggering and ineffective‐triggering than other masks that were not of the same brand. Also, the frequency of auto‐triggering and ineffective‐triggering was higher with a mask with more severe air leakage.

Both the Resmed Stellar™ 150 and the Curative Flexo ST30 ventilators do not typically have an oxygen control, and thus, supplemental oxygen is usually administered by adding it proximal to the mask or the ventilator.^18^ Our trial observed that differences in oxygen delivery position at the same oxygen flow rate or the same pressure support affected the frequency of auto‐triggering and ineffective‐triggering for the two different ventilators used in this trial. We speculate that this may be related to the different locations of the pressure sensors on the two different ventilators. This suggests that we should select the appropriate oxygen delivery position in clinical practice according to the type of ventilator used and the location of the sensor to ensure minimal abnormal triggering and improved patient–ventilator synchrony. Specifically, oxygen should be administered near the mask for the Resmed Stellar™ 150 ventilator and near the machine end for the Curative Flexo ST30 ventilator.

Compared with the ‘fully closed’ system of invasive mechanical ventilation, the characteristic of NIV is its ‘semi‐open’ system. Thus, air leaks around the mask are very likely to occur during NIV,^19^ that is, ‘non‐intentional’ leaks. Meanwhile, NIV has only a single limb circuit, so intentional gas leaks are deliberately required to expel exhaled gas and dilute exhaled carbon dioxide; those are ‘intentional’ leaks.^20^ Thus, gas leakage is inevitable with a non‐invasive ventilator, and air leakage is one of the most important factors affecting patient–ventilator synchrony.^21^, ^22^ Vignaux et al.^23^ conducted a bench model study that underlined that leaks entail major alterations in the key functions of ventilators and that NIVs exhibit substantial variability in their ability to cope with these problems.

We observed that there were only a few two‐by‐two comparisons with no statistical difference, and the frequency of auto‐triggering and ineffective‐triggering tended to increase with higher oxygen flow rates for both ventilators in the trial. Moreover, the frequency of auto‐triggering and ineffective‐triggering was smaller at lower oxygen flow rates (5 and 10 cm H_2_O), and the frequency of abnormal triggers was significantly higher at higher oxygen flow rates (15, 20 and 25 cm H_2_O). Higher oxygen flow may produce more unintentional air leaks, and we did not evaluate nonintentional leaks; thus, our trail speculated that oxygen flow can affect normal triggering of the ventilator. Meanwhile, the increase of abnormal triggering when increasing oxygen flow may be due to the impact of the added flow itself on the flow‐based trigger algorithms. It has been shown that expiratory leakage can mimic the inspiratory effort of a ventilator, leading to auto‐triggering,^24^ and inspiratory leaks can mimic a sustained inspiration, leading to delayed cycling^19^ and ineffective‐triggering. This suggests that we should pay close attention to the effectiveness of ventilators, which are used in patients if they need to be given high‐flow oxygen in clinical practice such as severe type I respiratory failure, and pay timely attention to the occurrence of patient–ventilator asynchrony.

We observed that there were only a few two‐by‐two comparisons with no statistical difference, and the frequency of auto‐triggering and ineffective‐triggering tended to increase with higher support pressures (IPAP and EPAP) for both ventilators in the trial. When IPAP and EPAP are raised to 16 cm H_2_O and 6 cm H_2_O, respectively, there is a very significant increase in the frequency of auto‐triggering and ineffective‐triggering. A study^9^ showed that 43% of patients presented with severe patient–ventilator asynchrony, and the two factors predictive of this phenomenon were the level of pressure support and the magnitude of leaks. Elevated levels of pressure support can increase the occurrence of asynchrony, which is consistent with our findings. Our findings suggest that in clinical practice, ventilators are more likely to be triggered abnormally if patients require a higher level of pressure support, so we should observe closely to detect the asynchrony and look for influencing factors so that the non‐invasive ventilator can be better used.

It has been known that interfaces used for ventilation could also affect patient–ventilator interaction,^20^ which is consistent with our findings. The C mask is a common mask in the clinic with severe air leakage. We found that different types of masks can affect the occurrence and frequency of auto‐triggering and ineffective‐triggering. We speculate that it may be related to the different airtightness of different material masks and the different sizes of masks, because different mask tightness and size can lead to different degrees of air leakage. Louis et al.^25^ investigated the effects of masks with various leakage levels on the performance of four ventilators while maintaining the same settings (ventilator‐recommended masks and masks with the largest and smallest leak levels). The results showed that the mask with the largest leakage level was associated with auto‐triggering and miss‐triggering with the majority of ventilators. Our findings suggest that we would better choose a mask whose brand is consistent with the brand of the ventilator. And when switching to a mask with a different leakage level, the trigger sensitivity of the ventilator must be rechecked to ensure patient comfort and ventilator efficacy.

Various limitations of this study should be outlined. Firstly, the subjects of this study were healthy adult volunteers with relatively uniform respiratory rates, tidal volumes and inspiratory capacities. Compared with the patients, the healthy volunteers tolerated the non‐invasive ventilator better at different oxygen flow rates and pressures and had a smoother and more cooperative mental state. Therefore, extrapolation of the results of this study to patients may be subject to some error. More clinical studies are needed to explore the effects of different disease states on abnormal triggering of the non‐invasive ventilator. Secondly, we studied only two types of non‐invasive ventilators that are representative in clinical practice, but we considered that differences in triggering may still exist between ventilator models, the rationale for which needs to be further validated. However, our aim in this study was not to describe the consistency of all devices but to explore the factors influencing abnormal triggering of non‐invasive ventilators through the above study.

In conclusion, when using different oxygen flow rates, pressure support levels, different oxygen injection sites and different types of masks, close attention should be paid to abnormal triggering of ventilators to minimize patient–ventilator asynchrony, improve patient comfort and ensure the effectiveness of therapy.

## ETHICS APPROVAL AND CONSENT TO PARTICIPATE

All procedures performed in studies involving human participants were in accordance with the 1964 Helsinki Declaration and its later amendments or comparable ethical standards. The trial was approved by the Medical Ethics Committee of Qilu Hospital of Shandong University (No. 2018216). Informed consent was obtained from all individual participants included in the study.

## CONSENT FOR PUBLICATION

Informed consent was obtained from all individual participants included in the study.

## CONFLICT OF INTEREST

The authors declare that they have no competing interests.

## AUTHOR CONTRIBUTIONS

ZN Xu and DQ Sheng performed the conceptualization. ZN Xu and DQ Sheng performed the methodology. ZN Xu, DQ Sheng and KF Jiao performed the formal analysis and investigation. ZN Xu, DQ Sheng, KF Jiao, C Zhang and JP Hao performed the data collection. ZN Xu, DQ Sheng, KF Jiao, C Zhang, JP Hao and DD Ma did the writing—original draft preparation. ZN Xu, DQ Sheng, KF Jiao, C Zhang, JP Hao and DD Ma did the writing—review and editing. DD Ma did the funding acquisition. DD Ma performed the supervision.

## Data Availability

The data that support the findings of this study are available from the corresponding author upon reasonable request.
